# Complete Genome Sequence of the New Urolithin-Producing Bacterium *Gordonibacter urolithinfaciens* DSM 27213^T^

**DOI:** 10.1128/genomeA.01120-17

**Published:** 2017-12-14

**Authors:** Juan F. Martinez-Blanch, Daniel Ramón, David Beltrán, María Romo-Vaquero, Rocío García-Villalba, Juan C. Espín, Francisco A. Tomás-Barberán, Francisco M. Codoñer, María V. Selma

**Affiliations:** a Lifesequencing SL, Paterna, Valencia, Spain; b Biópolis SL, Paterna, Valencia, Spain; c Laboratory of Food and Health, Research Group on Quality, Safety and Bioactivity of Plant Foods, Department of Food Science and Technology, CEBAS-CSIC, Murcia, Spain

## Abstract

*Gordonibacter urolithinfaciens* DSM 27213^T^ was isolated from human feces and is able to metabolize ellagic acid (a dietary phenolic compound present in various fruits) to urolithins. Here, we report the finished and annotated genome sequence of this organism.

## GENOME ANNOUNCEMENT

The potential health benefits of polyphenol-rich foods have become increasingly recognized worldwide. Ellagitannins are polyphenols present in berries (strawberry, raspberry, blackberry, and cloudberry, among others), pomegranates, muscadine grapes, walnuts, almonds, and oak-aged wines, among other foods, with the principal component being ellagic acid (EA). EA and ellagitannins are metabolized to anti-inflammatory, cardioprotective, and anticancer metabolite urolithins by the gut microbiota (1). *Gordonibacter urolithinfaciens* DSM 27213^T^ was isolated from the feces of a healthy human and is able to metabolize ellagic acid to urolithins (2, 3). Here, we present the complete genome sequence of this strain.

Complete genome sequencing of the *Gordonibacter urolithinfaciens* DSM 27213^T^ strain was carried out using the high-throughput sequencing technology as implemented using the PacBio RSII platform (Pacific Biosciences, Menlo Park, CA). A 10-kbp insert library was constructed with purified DNA, and two single-molecule real-time (SMRT) cells were sequenced using XL-C2 chemistry and a data collection time of 180 min. The sequencing run provided a total amount of 300,584 sequences, with an accuracy of Q20. The obtained sequences were filtered by quality, and a total of 120,294 sequences were obtained, with a mean length of 10,209 nucleotides (nt) of quality Q20. The total data output was 1.228 Gb. A *de novo* assembly employed the default parameters within the Hierarchical Genome Assembly Process 3 (HGAP3, PacBio DevNet; Pacific Biosciences) approach. Finally, 114,420 reads of around 8.9 kb were obtained, providing a unique circular contig of 3,294,868 bp (GC content, 66%) and coverage of 286×. No plasmids were detected.

Genome annotation was performed using Prokka annotation pipeline (version 1.11 [4]), which involves predicting tRNA, rRNA, and mRNA genes and signal peptides in the sequences using ARAGORN, RNAmmer, Prodigal, and SignalP, respectively (5–8). The genome contains 2,841 elements, of which 2,788 are open reading frames (ORFs) (2,369 canonical and 419 noncanonical). Of the 2,841 elements, 62 are structural RNAs (sRNAs) (9 rRNAs and 53 tRNAs). To search in depth for similarities between the DSM 27213^T^ strain and the phylogenetically nearest strain, *Gordonibacter pamelaeae* DSM 19378^T^ (genome, GenBank accession no. FP929047), the genome sequences were compared. We found that the DSM 27213^T^ genome contains 471 well-annotated elements unique to this strain that are missing in strain DSM 19378^T^. In this respect, an analysis of the complete genome of *G. urolithinfaciens* DSM 27213^T^ may help us understand the mechanisms involved in metabolization of ellagic acid to urolithins.

### Accession number(s).

The microbial strain was deposited at the German Collection of Microorganisms and Cell Cultures (DSMZ) with the deposit number DSM 27213^T^. The results of the whole-genome project have been deposited at DDBJ/EMBL/GenBank under accession no. LT900217. The version described here is the first version.
